# GRASLND regulates melanoma cell progression by targeting the miR-218-5p/STAM2 axis

**DOI:** 10.1186/s12967-024-05397-z

**Published:** 2024-07-26

**Authors:** Aiwei Ma, Wenqi Shi, Liyun Chen, Zijian Huang, Yiwen Zhang, Zixuan Tang, Wenshi Jiang, Mengjing Xu, Jianda Zhou, Wancong Zhang, Shijie Tang

**Affiliations:** 1 Department of Plastic Surgery and Burns Center, Second Affiliated Hospital, Shantou University Medical College, Shantou, Guangdong 515051 China; 2 Plastic Surgery Institute of Shantou University Medical College, Shantou, Guangdong 515051 China; 3 Shantou Plastic Surgery Clinical Research Center, Shantou, Guangdong 515051 China; 4 Research Center of Translational Medicine, Second Affiliated Hospital of Shantou University Medical College, Shantou, Guangdong 515051 China; 5 Department of Plastic and Reconstructive Surgery, Central South University Third Xiangya Hospital, Changsha, China

**Keywords:** SKCM, lncRNA, Prognosis, Immunotherapy, Biomarker

## Abstract

**Background:**

Increasing evidence suggests that long noncoding RNAs (lncRNAs) play important regulatory roles in biological processes and are dysregulated in numerous tumors. The lncRNA GRASLND functions as an oncogene in many cancers, but its role in skin cutaneous melanoma (SKCM) requires further investigation.

**Methods:**

SiRNA transfection, wound − healing and transwell assays were performed to evaluate the effect of GRASLND on cellular function.

**Results:**

The present study demonstrated that GRASLND expression is increased in SKCM tissues and cell lines. The high expression of GRASLND was correlated with poor prognosis and immunotherapy outcomes. Knockdown of GRASLND significantly inhibited cell migration and invasion. In addition, we found that miR-218-5p directly binds to its binding site on GRASLND, and GRASLND and miR-218-5p demonstrate mutual inhibition. Furthermore, the miR-218-5p inhibitor partially eliminated the knockdown of GRASLND and inhibited its expression. We also demonstrated that GRASLND acts as a miR-218-5p sponge that positively regulates STAM2 expression in SKCM cells.

**Conclusion:**

In summary, these data suggest that GRASLND functions by regulating miR-218-5p/STAM2 expression, suggesting an important role for the lncRNA‒miRNA–mRNA functional network and a new potential therapeutic target for SKCM.

## Introduction

Skin melanoma (SKCM) is a common skin tumor causing ~ 55,500 deaths annually [1]. Melanoma accounts for approximately 1.7% of all newly diagnosed primary malignant cancers worldwide, and melanoma-related deaths account for ~ 0.7% of all cancer-related deaths [1]. In recent years, the incidence of SKCM has increased annually, imposing a substantial social and economic burden [2]. The main current treatment for SKCM is surgery, but even with surgical resection, the prognosis remains poor. The 5-year survival rates for patients with limited, regional, and metastatic SKCM were 98%, 64%, and 23%, respectively [3]. This poor survival rate is partially because the disease is challenging to diagnose in the early stage and less curable in the late stage. Therefore, screening for potential molecular diagnostic markers and therapeutic targets is essential for predicting patient prognosis and developing personalized treatments.

Long noncoding RNAs (lncRNAs) are a class of RNA molecules greater than 200 nucleotides in length that are transcribed from genes but not translated into proteins. Initially, lncRNAs were thought to be “junk sequences” that did not perform important functions. Recent studies have shown that lncRNAs are involved in tumorigenesis by maintaining proliferative signals, activating invasion and metastasis, and inducing angiogenesis (Table 1) [4–22]. LncRNAs also play important roles in SKCM. For example, silencing the lncRNA SAMMSON inhibits melanoma survival by disrupting important p32-mediated mitochondrial functions [23]. In addition, the lncRNA SLNCR1 increases melanoma invasion by transcriptionally upregulating matrix metalloproteinase 9 (MMP9) through cooperation with brain-specific homologous box protein 3a (Brn3a) and the androgen receptor (AR) [24]. Iyer et al. [25] identified 339 melanoma-associated lncRNAs; however, the effects of most lncRNAs on melanoma are currently unknown.

**Table 1:** The role of lncRNAs in various cancers

Cancer	lncRNAs	Function	Role	Reference	
Bladder cancer	RP11-89	Proliferation, migration	Oncogene	[5]	
	KCNQ1OT1	Proliferation, metastasis	Oncogene	[6]	
Liver cancer	LUCAT1	Proliferation, metastasis	Oncogene	[7]	
	TLNC1	Metastasis	Oncogene	[8]	
Gastric cancer	TP53TG1	Proliferation, metastasis, cell cycle	Tumor suppressive	[9]	
	MIR200CHG	EMT	Tumor suppressive	[10]	
Colorectal cancer	BCAR4	Proliferation, migration	Oncogene	[11]	
	HOTAIR	Metastasis	Oncogene	[12]	
Breast cancer	PCAT19	Proliferation	Tumor suppressive	[13]	
	SNHG5	Angiogenesis	Oncogene	[14]	
Lung cancer	LINC02159	Proliferation, migration, invasion	Oncogene	[15]	
	SLCO4A1-AS1	Migration, invasion	Tumor suppressive	[16]	
Prostate cancer	NEAT1	Bone metastasis	Oncogene	[17]	
	RP11-1023L17.1	Proliferation, migration, cell cycle	Oncogene	[18]	
Ovarian cancer	MALAT1	Invasion	Oncogene	[19]	
	SPOCD1-AS	Metastasis	Oncogene	[20]	
Cervical cancer	SFTA1P	Tumorigenesis, metastasis	Oncogene	[21]	
	PTENP1	Proliferation, EMT	Tumor suppressive	[22]	

In this study, we screened a series of differentially expressed lncRNAs between SKCM and healthy skin tissues via gene chip and The Cancer Genome Atlas Program (TCGA )RNA-seq data integration analyses, constructed a model via machine learning algorithms, and further downscaled the model to obtain the key lncRNA GRASLND. Subsequently, we performed cellular experiments and utilized multiorganomics data mining to explore the functions, mechanisms, and clinical biomarkers of the lncRNAs. Our findings suggest that GRASLND is a potential marker for prognostic and immunotherapeutic response determination in SKCM patients, and an in-depth study of its functional mechanism may provide new targets and therapeutic strategies for melanoma immunotherapy.

## Methods and materials

### Tissue sample collection

Melanoma (*n* = 16) and paraneoplastic normal (*n* = 12) tissue specimens were obtained from patients with cutaneous melanoma who underwent tumor resection at the Third Xiangya Hospital of Central South University. The histologic type of our selected case was nevoid melanoma. The study was approved by the Ethics Committee of the Third Xiangya Hospital of Central South University, and informed consent was obtained from all the participants.

### Cell culture and siRNA transfection

Two melanoma cell lines, A2058 and SK-MEL-28, were purchased from the American Type Culture Collection (ATCC). A2058 and SK-MEL-28 cells were cultured in Dulbecco’s modified Eagle’s medium (DMEM)(Gibco, USA) and Roswell Park Memorial Institute 1640 medium(RPMI-1640)(Gibco, USA), respectively. All cells were cultured in a medium containing 10% fetal bovine serum (FBS) (Gibco, USA) and 1% 100× penicillin-streptomycin (Beyotime Biotechnology, China) and incubated at 37 °C under 5% CO2 until adherent growth. Hieff Trans® in vitro siRNA/miRNA Transfection Reagent Transfection Kit (Yeasen, China) was used in strict accordance with the instructions provided. The GRASLND siRNA and nontargeting negative control (NC) used in this study were obtained from Gemma Genetics with the following sequences: si-918, GGCAAGAUAAAUGACAAUAAATT, and si-1208, GUGGGAGUAGCAUCCACAUAATT.

### qRT‒PCR

Total RNA was extracted from the cells using TRIzol Reagent (Yeason, China) and reverse transcribed using 5× Hifair® One Step RT SuperMix (Yeason, China). cDNA was used as a template for amplification using SYBR Green PCR mix reagent (Yeason, China). The primers used were synthesized by Guangzhou Aiki Biotechnology Co. The sequences of primers used were as follows: GRASLND, forward, 5′-AGGATTCAGGGGATGCACAG-3′ and reverse, 5′-TGGGCTGAAGATGAGACGTT-3′; GAPDH, forward, 5’-TGCACCACCAACTGCTTAGC-3’; and reverse 5’-GGCATGGACTGTGGTCATGAG-3’. GAPDH was used as a reference to normalize GRASLND expression. The relative expression of GRASLND was calculated using the 2-ΔΔCt method. Each quantitation of mRNA levels represents data from three independent experiments.

#### Transwell migration

After target gene silencing or overexpression, the cells were grouped and inoculated in 8 μm wells in the upper chamber of a Transwell plate (Corning, NY). The upper chamber of the Transwell system was supplemented with 200 µl of serum-free DMEM, and the lower chamber was supplemented with 600 µl of 10% FBS DMEM. After 48 h of incubation at 37 °C with 5% CO^2^, each well was washed twice with PBS. The migrated cells were then fixed with fixative and stained with 0.1% crystal violet. Six fields of view were randomly photographed under a microscope, and the migrated cells were counted.

### Wound-healing assay

The monolayer was then scratched with a sterile 20 µl plastic pipette tip, and the detached cells were removed with PBS. The cells were cultured in low-serum DMEM or RPMI 1640 medium to inhibit cell proliferation, and images were captured using an inverted microscope (ZEISS, Axio Observer A1) at the indicated times (0, 12, 24, and 48 h).

### Fluorescence in situ hybridization (FISH)

The probes used in this study were designed and synthesized by RiboBio (Guangzhou, China). Fluorescence in situ hybridization (FISH) was performed using a FISH kit (RiboBio, China) following the manufacturer’s instructions. 6 × 10^4^ cells were seeded 24-well plate with cover glass and cultured to 60-70% density. The cells were fixed using paraformaldehyde and permeabilized with triton x-100. Subsequently, 20 µl of prehybridization solution was added to each well, and then 200 µl of hybridization solution containing the probe was added. The mixture was hybridized overnight at 37 °C in the dark. The hybridization solution was removed, and the cells were washed, keyed, sealed with an anti-fluorescence quencher, and photographed using a laser confocal microscope (Zeiss, Germany).

### Dual luciferase assay

GRASLND-WT (wild-type), GRASLND-MUT (mutant), STAM2-WT (wild-type), and STAM2-MUT (mutant) melanoma cells were cultured in 6-well plates and transfected with the miR-218-5p mimic or control siRNA. 48 h after transfection, we assessed the luciferase activity of the firefly and sea pansy (Renilla) strains using the Dual-Luciferase Reporter Assay System (Promega). Relative luciferase activity was calculated from the ratio of firefly/sea pansy (Renilla) luciferase activity and normalized to that of the control. The firefly luciferase activity of each transfected well was normalized to the Renilla luciferase activity. Each assay was performed in triplicate.

### Protein isolation and Western blotting

Total protein was extracted from melanoma cells using RIPA extraction reagent (Shanghai Biyuntian Company, China) and a protease inhibitor mixture (Shanghai Biyuntian Company, China). Proteins were separated by 10% SDS‒PAGE and then electroblotted onto a polyvinylidene fluoride (PVDF) membrane (Millipore). The membranes were blocked with 5% skim milk for 1 h at room temperature and incubated overnight at 4 °C with various specific primary antibodies. The next day, the blots were washed and incubated with horseradish peroxidase-conjugated secondary antibodies (Millipore) for 2 h. The protein bands were detected using the ECL Chemiluminescent Substrate Reagent (Biosharp, China). β-Actin served as a loading control.

### Statistical analysis

Statistical analyses were performed using R language and GraphPad Prism 8.0.2 software. Student’s t-test was used to determine the significance of differences between the two groups. Significant differences between multiple groups were calculated using analysis of variance (ANOVA). Survival curves were generated via Kaplan‒Meier analysis and the log-rank test. Correlation analysis was performed using Pearson’s correlation coefficient. *p* < 0.05 was considered to indicate significance.

## Results

### GRASLND is highly expressed in melanoma

**Fig. 1:**
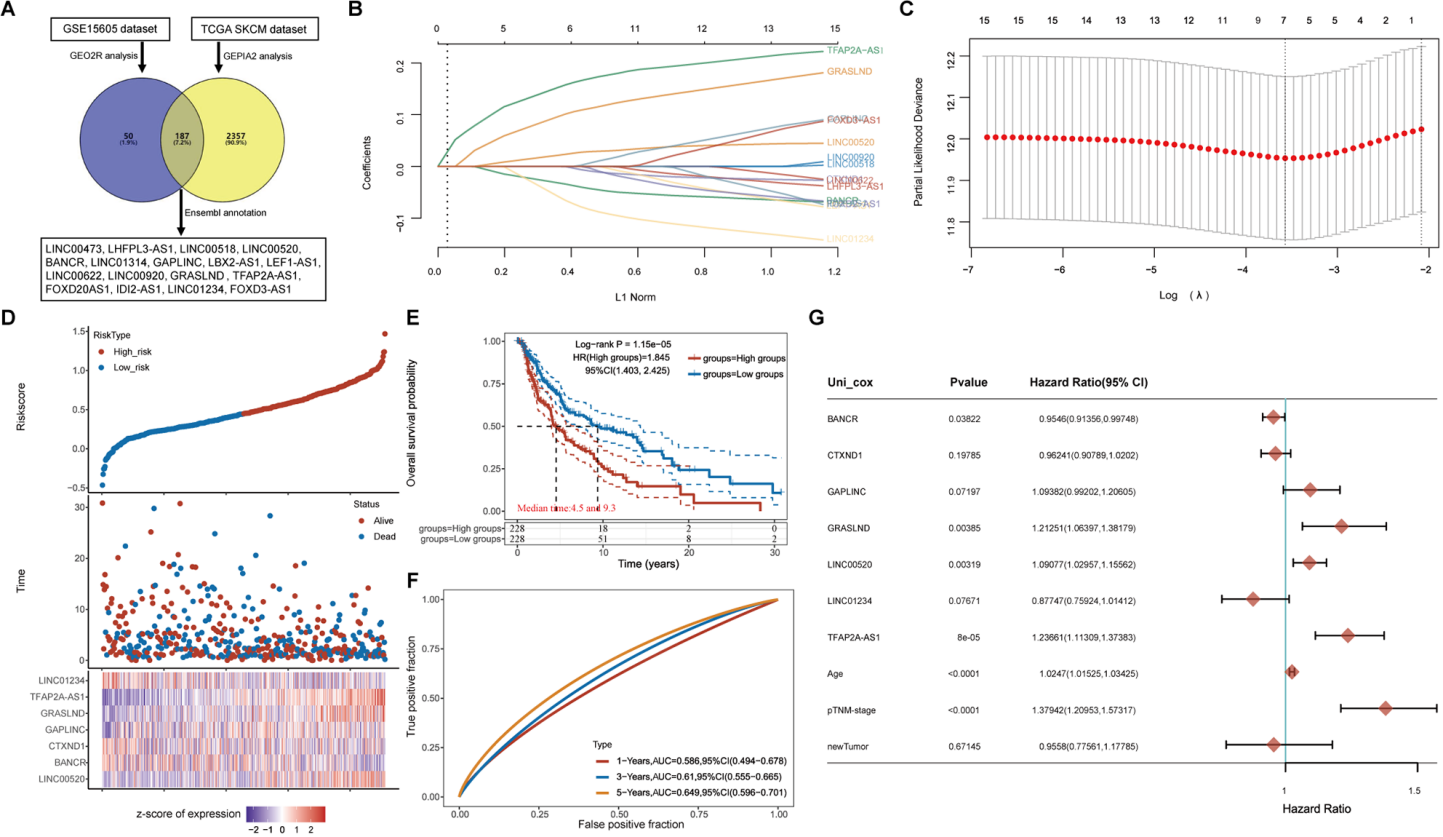
Construction of the marker lncRNA prognostic model. (**A**) Seventeen lncRNAs differentially expressed in the GSE15605 dataset and TCGA cohort. (**B**) Graph of the LASSO coefficient distribution of marker lncRNAs in GSE15605. (**C**) Graph of the tenfold cross-validation error rate. (**D**) Distribution of risk scores (upper panel), survival statuses (middle panel), and expression characteristics of 7 prognosis-related lncRNAs in SKCM. (**E**) Survival curves comparing the overall survival probability of melanoma patients in the low-risk versus high-risk groups. (**F**) ROC curves predicting the risk of death at 1, 3, and 5 years in melanoma patients. (**G**) One-way Cox regression analysis of prognostically relevant lncRNAs in 7 patients

In this study, we first obtained the differentially expressed RNAs from the GSE15605 dataset via the GEO2R online tool. Subsequently, we obtained the differentially expressed RNAs from the TCGA dataset using the GEPIA2(http://gepia2.cancer-pku.cn/#index) online tool. Next, we analyzed the intersection of these two sets of differentially expressed RNAs and used the Ensembl database to obtain the shared differentially expressed RNAs annotated with lncRNAs. Through this series of analyses, we found 187 differentially expressed RNAs, 17 of which were lncRNAs (Fig. 1A). Based on these 17 lncRNAs, we used The least absolute shrinkage and selection operator (LASSO) regression analysis to construct a risk score model consisting of 7 lncRNAs (LINC00520, BANCR, GAPLINC, TFAP2A-AS1, LINC01234, GRASLND, and CTXND1) (Fig. 1B, C). Kaplan‒Meier curves revealed a significant difference in survival between the high-risk and low-risk patients (*p* < 0.05), while the 1–3–5 year receiver operating characteristic (ROC) curves of the risk model and the area under the curve (AUC) analysis indicated the strong predictive ability of the model (Fig. 1D-F). These results confirmed the high accuracy and reliability of the lncRNA-based risk score model we constructed for predicting the survival status of SKCM patients.

To further evaluate the clinical significance of these seven lncRNAs in cutaneous melanoma, we performed a one-way Cox regression analysis. The results showed that the hazard ratios (HRs) of BANCR, CTXND1, GAPLINC, LINC00520, LINC01234, TFAP2A-AS1, and GRASLND were 0.9546, 0.96241, 1.21251, 0.87747, 1.23661, 1.0247, and 1.09077, respectively (Fig. 1G). Combined with the *p* values, the trends of two of these molecules, GRASLND and TFAP2A-AS1, were in good agreement with the model, and we subsequently analyzed these molecules in depth.

### High expression of GRASLND in melanoma is significantly associated with poor prognosis

**Fig. 2:**
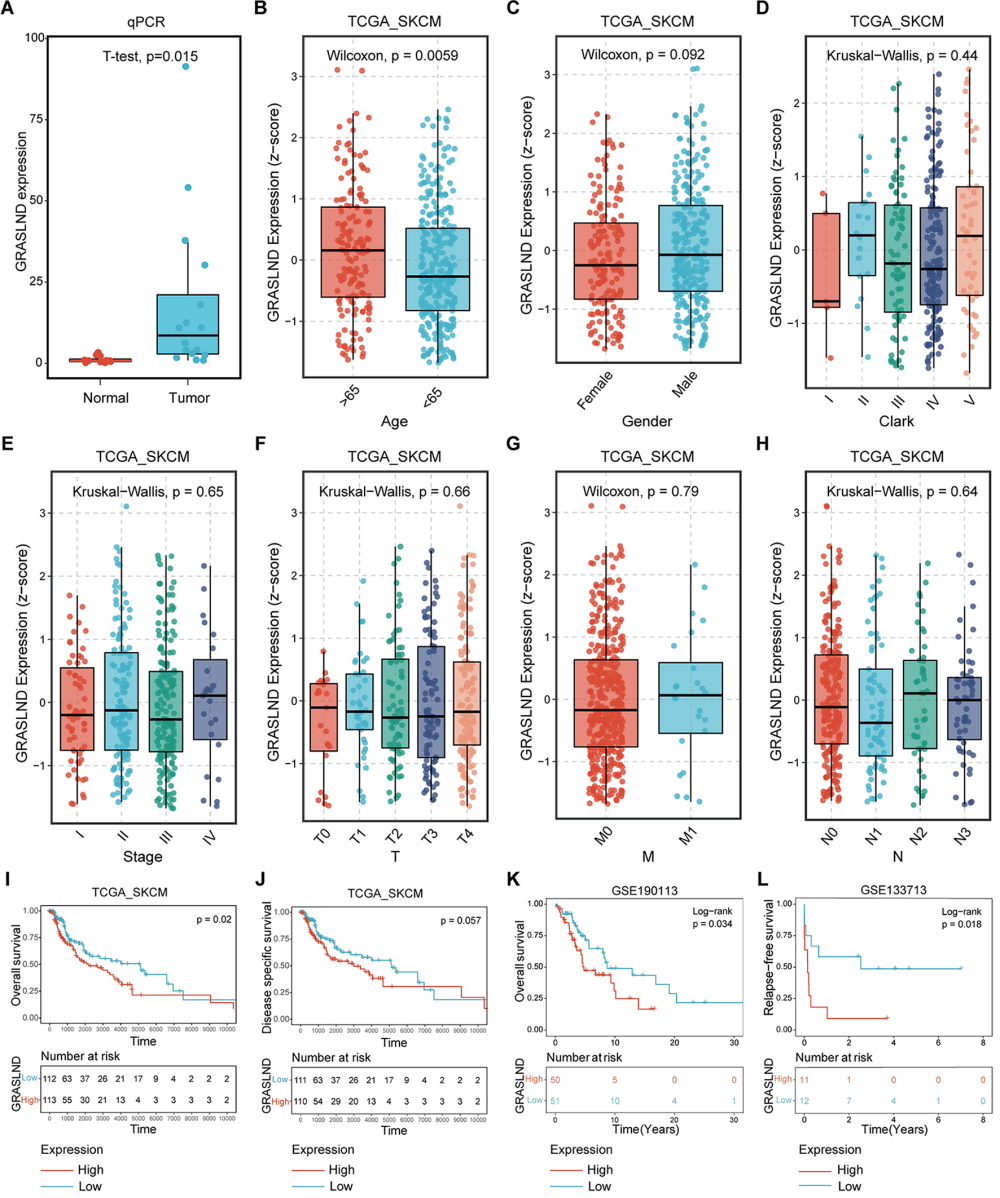
Prognostic value of GRASLND in melanoma patients. (**A**) Differential expression of GRASLND in normal and SKCM tumor tissues measured by qRT–PCR. (**B-H**) Differential expression of GRASLND between different age groups, sexes, Clark staging periods, clinical staging periods, T staging periods, M staging periods, and N staging periods. (**I, J**) TCGA data were analyzed to evaluate the correlation between the expression of GRASLND and OS/DSS. (**K, L**) Correlations of GRASLND expression with OS/RFS in different datasets

To further validate the two molecules screened in the prescreening, we first assessed the expression level of GRASLND in melanoma and normal tissues by qRT–PCR, and the results showed that its expression was significantly upregulated in SKCM (Fig. 2A). In addition, we analyzed the relationship between GRASLND in the TCGA SKCM cohort and the clinical characteristics of the patients. GRASLND expression was significantly greater in the group of patients older than 65 years than in the group of patients younger than 65 years (Fig. 2B). However, the expression of GRASLND has no difference for any of the clinical characteristics, including sex, Clark stage, clinical stage, T stage, M stage, and N stage (Fig. 2C-H).

To further explore the relationship between GRASLND expression levels and survival outcomes in patients with SKCM, we first analyzed the relationship between GRASLND expression and the corresponding prognostic information using SKCM cohort data from the TCGA database. The overall survival (OS) analysis revealed that patients with high GRASLND expression had lower survival rates (Fig. 2I). However, disease-specific survival (DSS) analysis showed that survival was not significant in patients with high GRASLND expression levels compared to those with low expression (Fig. 2J). These results suggest that high GRASLND expression levels are strongly associated with poor prognosis in melanoma patients. Finally, we used two external datasets, GSE190113 and GSE33713, to analyze the correlation between the expression level of GRASLND and OS and relapse-free survival (RFS) outcomes. Consistent with the aforementioned TCGA SKCM cohort data, in the GSE190113 dataset, the survival analyses revealed that patients with high GRASLND expression had poor OS (Fig. 2K). The GSE33713 dataset analysis revealed that patients with high GRASLND expression also exhibited poor RFS (Fig. 2L). These results further support the association between GRASLND expression and poor prognosis.

### The expression of GRASLND negatively correlates with immune infiltration and immune molecules expression

**Fig. 3:**
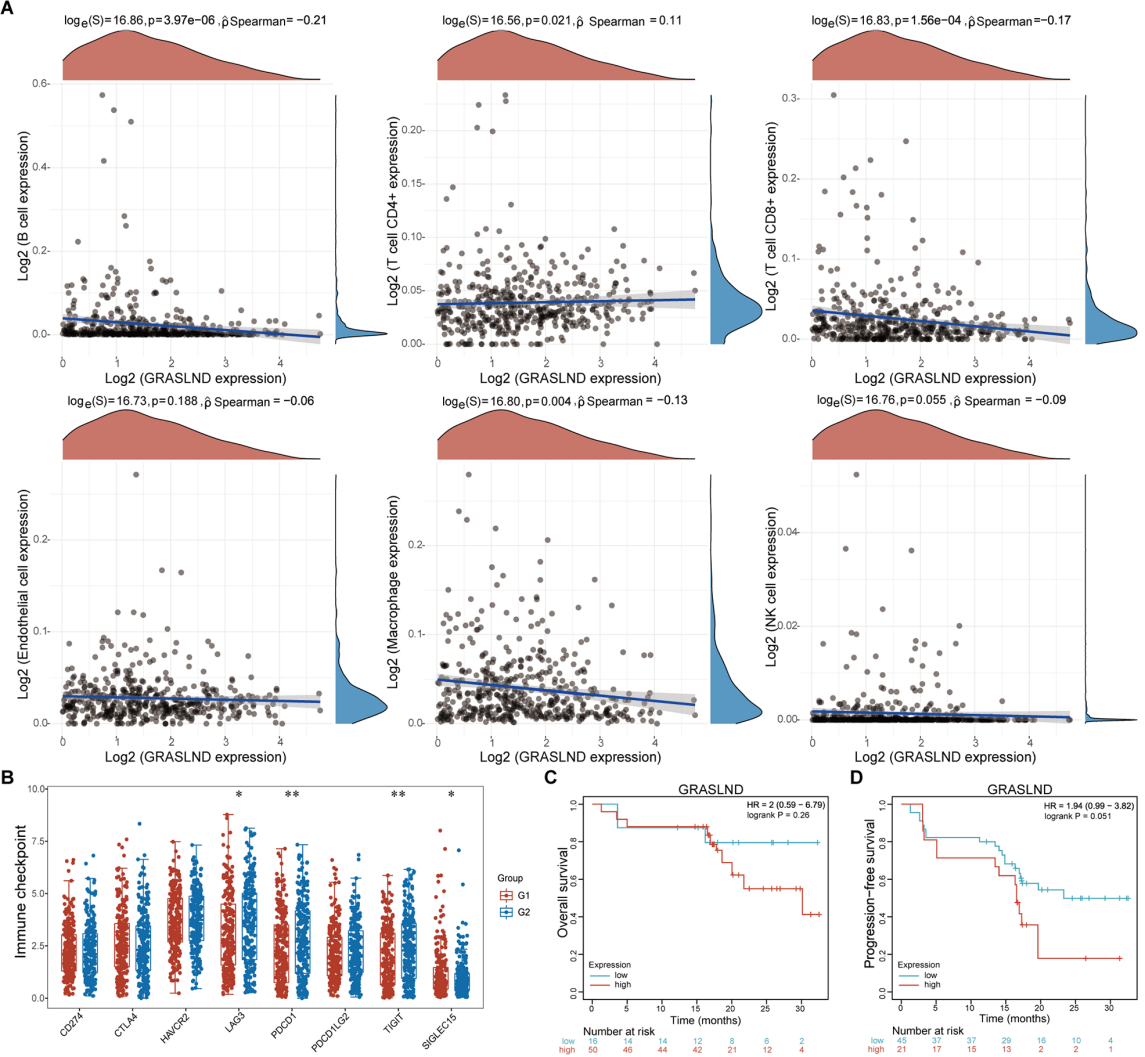
Correlations of GRASLND expression with immune infiltration and immunotherapy outcomes. (**A**) Correlation analysis of GRASLND expression with the abundances of different immune cells. (**B**) Comparison of the expression levels of immune checkpoint molecules in the GRASLND high expression (G1) and low expression (G2) groups. (**C, D**) Correlation between GRASLND expression and OS/PFS outcomes

To further investigate the relationship between GRASLND expression and immune cell infiltration, we calculated the correlation between GRASLND expression and the abundance of various immune cells (e.g., B cells, CD4 + T cells, CD8 + T cells, endothelial cells, macrophages, and NK cells). The results showed a negative correlation between GRASLND expression and CD8 + T-cell abundance. This finding suggested that GRASLND expression may also affect the infiltration of this immune cell subset (Fig. 3A). We further investigated the relationship between GRASLND expression and the immune checkpoint molecules expression(e.g., LAG3, PDCD1, and TIGIT). We compared the expression levels of immune checkpoint molecules in the high GRASLND expression (G1) and low GRASLND expression(G2) groups and used independent two-sample t-tests to assess the differences between the two groups. We found that LAG3 expression was significantly greater in the G1 subgroup than in the G2 subgroup, whereas the expression of PDCD1 and TIGIT was significantly lower in the G2 subgroup than in the G1 subgroup. These results suggest that GRASLND expression may impact the expression of immune checkpoint molecules, thereby modulating the function of immune cells in the tumor microenvironment (Fig. 3B). Furthermore, we found that patients with high GRASLND expression had lower OS and PFS rates (Fig. 3C-D). In conclusion, our analysis revealed a negative correlation between GRASLND expression immune cell infiltration, and immune checkpoint molecule expression.

### GRASLND significantly promotes SKCM cell migration

**Fig. 4:**
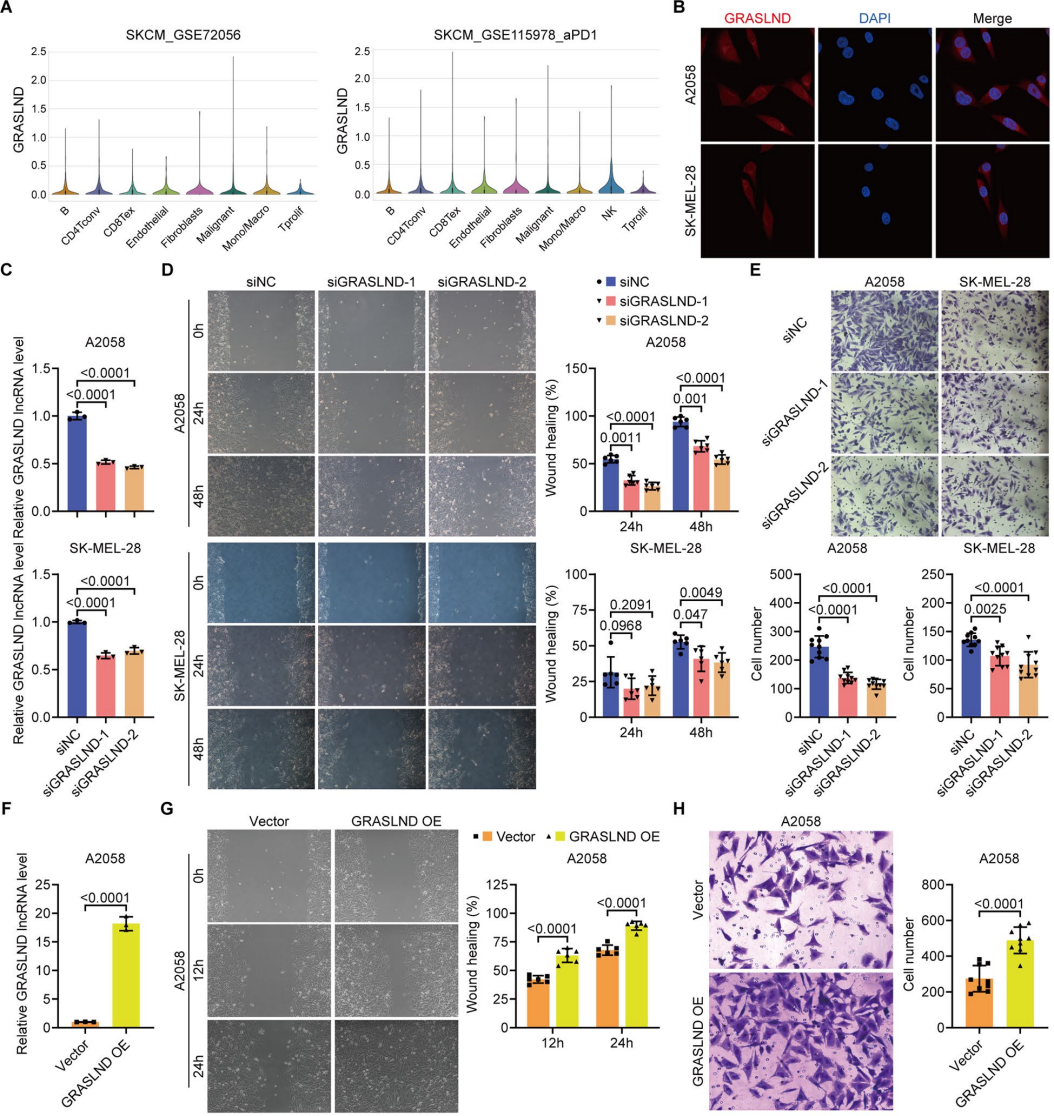
Construction of the marker lncRNA prognostic model. (**A**) Expression levels of GRASLND in various cells in the GSE72056 and GSE115978_aPD1 single-cell transcriptome datasets. (**B**) Fluorescence in situ hybridization showed that GRASLND was expressed both in the nucleus and cytoplasm of A2058 and SK-MEL-28 cells. (**C**) Transfection of siRNA or NC targeting GRASLND and measurement of GRASLND expression levels in A2058 and SK-MEL-28 cells by qRT‒PCR. (**D**) The wound healing assay showed that the knockdown of GRASLND inhibited the migration ability of A2058 cells and SK-MEL-28 cells. (**E**) Migration assays showed that GRASLND knockdown significantly inhibited the migration of A2058 and SK-MEL-28 cells. (**F**) After overexpressing GRASLND, the expression level of GRASLND in A2058 cells was measured by qRT‒PCR. (**G**) The wound healing assay showed that the migration ability of A2058 cells was enhanced after overexpression of GRASLND. (**H**) Migration assays showed that overexpression of GRASLND significantly enhanced the migration ability of A2058 cells

To determine the cell types expressing GRASLND, we extracted the expression levels of GRASLND in cell subpopulation species from two datasets, GSE72056 and GSE115978_aPD1. The results showed that GRASLND was expressed in eight cell types in both datasets: B cells, CD4 + T cells, CD8 + T cells, endothelial cells, fibroblasts, malignant cells, monocytes/macrophages, and value-added T cells (Fig. 4A). The localization of GRASLND in A2058 and SK-MEL-28 was examined by FISH. The results showed that GRASLND was located mainly in the nucleus and partially in the cytoplasm (Fig. 4B). We used two siRNA(si-918 and si-1208) to knock down GRASLND in SKCM cells (Fig. 4C). Silencing of GRASLND decreased the wound healing rate, indicating that silencing of GRASLND inhibited cell migratory capacity (Fig. 4D). In the Transwell assays, GRASLND silencing significantly reduced the number of migrating cells (Fig. 4E). We also constructed a GRASLND overexpression model by lentiviral transduction in A2058 cells (Fig. 4F). In agreement with the findings of the previous experiments, GRASLND overexpression significantly enhanced the migratory ability of the cells (Fig. 4G, H). These findings suggest that GRASLND plays an important role in promoting the migration of SKCM cells.

### GRASLND functions as a sponge of miR-218-5p

**Fig. 5:**
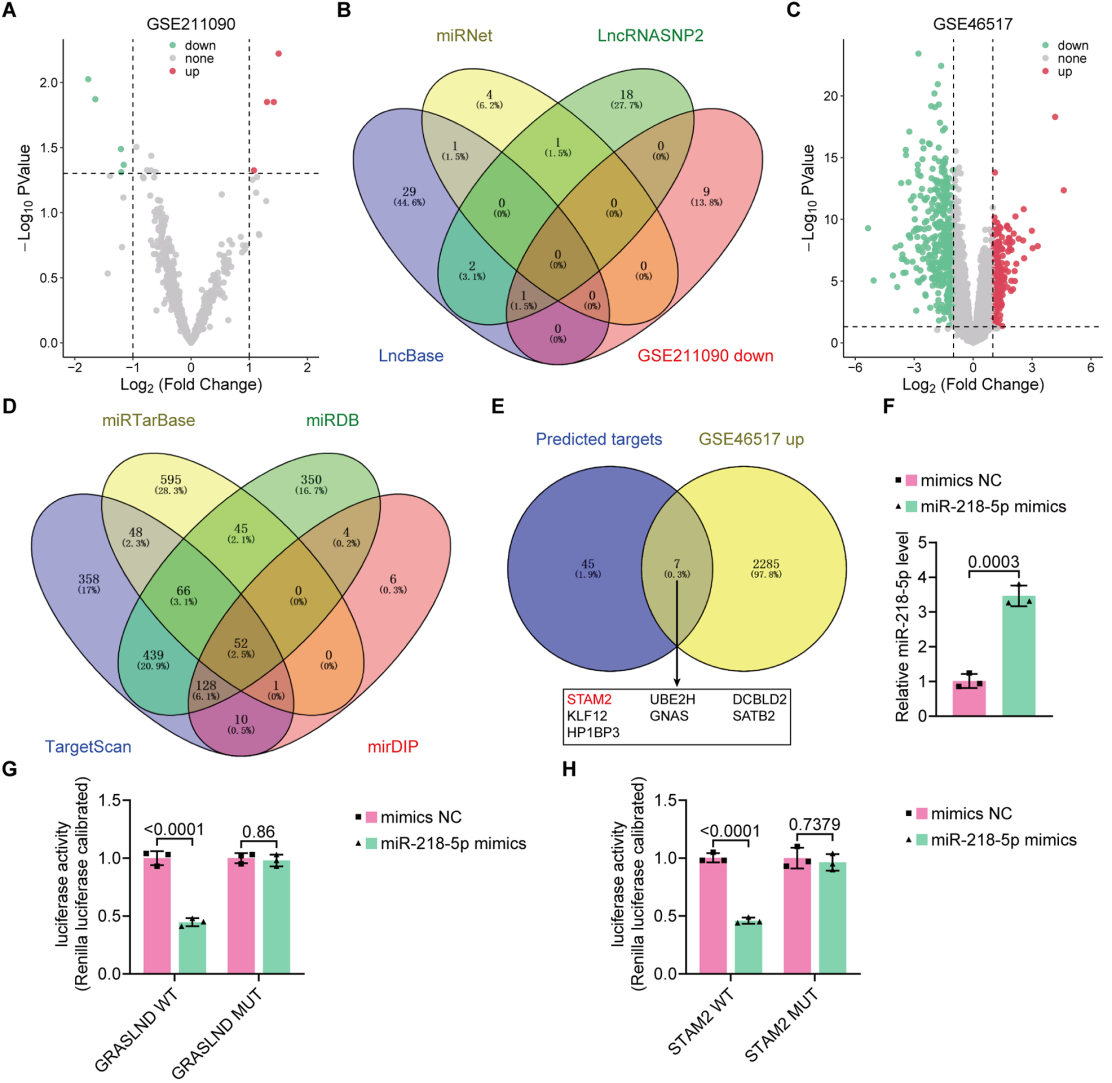
Flow chart of the screening of miRNAs and target genes associated with GRASLND. (**A, C**) Volcano plots of differentially expressed genes. (**B, D**) Venn diagram of differential gene expression. (**E**) Flowchart showing target gene annotation. (**F**) Transfection of mimics or NC targeting miR-218-5p and measurement of miR-218-5p expression levels in cells by qRT‒PCR. (**G, H**) A dual-luciferase reporter gene assay was used to measure luciferase activity in SK-MEL-28 cells

After identifying GRASLND as an oncogene in SKCM, our next goal was to determine the underlying mechanisms involved. Considering the cytoplasmic localization of GRASLND in SKCM cells, we used online tools such as TargetScan 7.2 (http://www.targetscan.org/vert_72/) and the GSE database to predict the potential binding sites of miRNAs to GRASLND (Fig. 5A-D). We detected elevated miR-218-5p expression after transfection of the NC or miR-218-5p mimics (Fig. 5F). Subsequently, we specifically explored the relationship between miR-218-5p and GRASLND.

We mutated the binding sequence of miR-218-5p to GRASLND and cotransfected SK-MEL-28 cells with reporter vectors carrying the wild-type GRASLND sequence, effectively reducing luciferase activity. However, mutation of the miR-218-5p binding site eliminated the inhibitory effect of the miR-218-5p mimic on the GRASLND- and STAM2-driven luciferase activities (Fig. 5G, H).

### GRASLND positively regulates STAM2 expression by sponging miR-218-5p

**Fig. 6:**
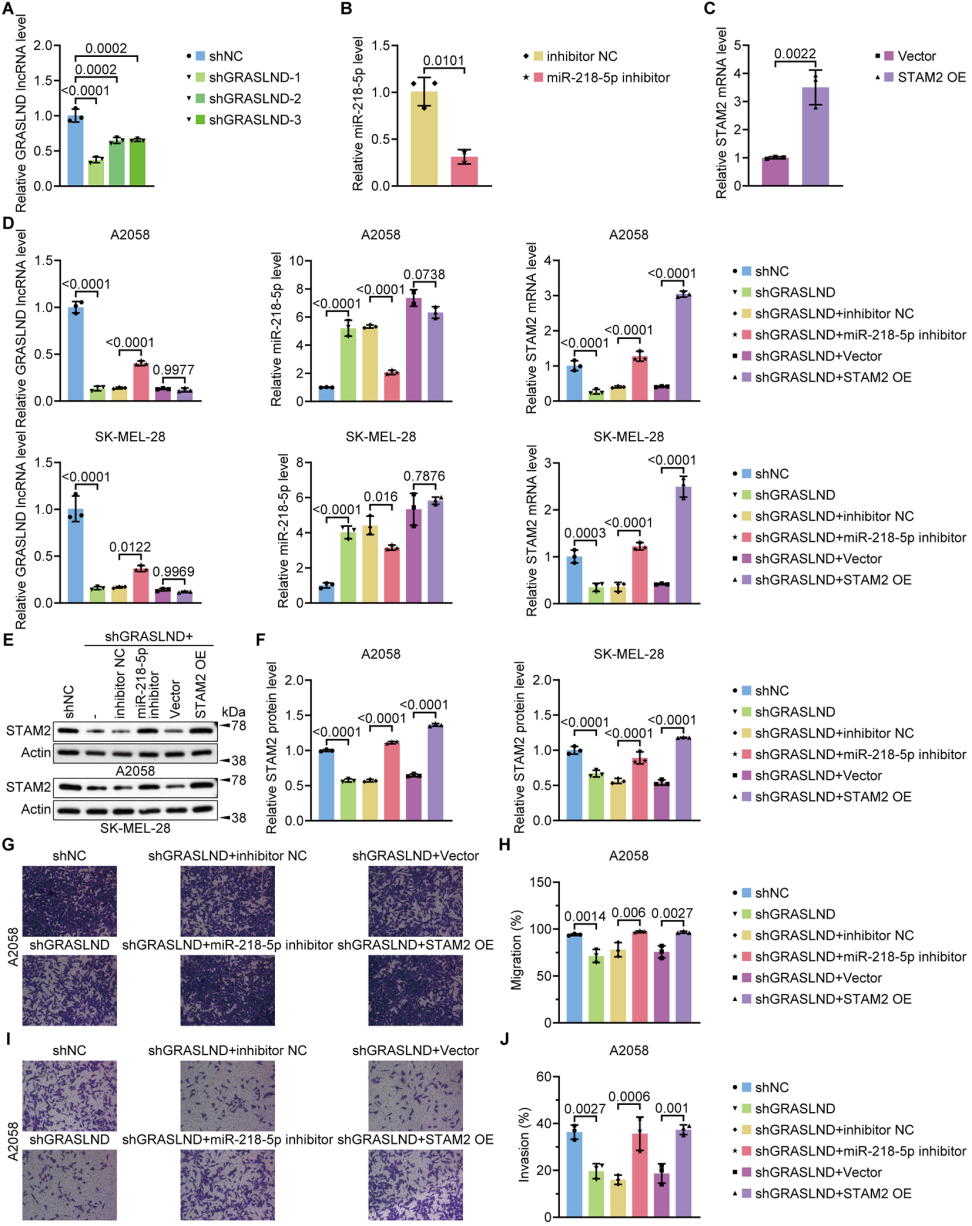
GRASLND affects the migration and invasion of SKCM cells by regulating miR-218-5p expression. (**A**) After silencing GRASLND, qRT‒PCR was performed to measure GRASLND expression. (**B**) After transfection of cells with the miR-218-5p inhibitor or NC, qRT‒PCR was performed to measure miR-218-5p expression. (**C**) After overexpressing GRASLND, qRT‒PCR was performed to measure GRASLND expression. (**D, E**) The RNA and protein expression levels of GRASLND, miR-218-5p, and STAM2 in A2058 cells and SK-MEL-28 cells were measured by qRT‒PCR and Western blotting after the transfection of NC or shGRASLND and after the cotransfection of shGRASLND with the miR-218-5p inhibitor. (**F**) Grayscale statistics of the protein expression of STAM2 in A2058 cells and SK-MEL-28 cells after transfection with NC or shGRASLND and cotransfection of shGRASLND with the miR-218-5p inhibitor. (**G-J**) Effect on the migration and invasion of A2058 cells after transfection with NC or shGRASLND or cotransfection of shGRASLND with the miR-218-5p inhibitor

To further explore whether GRASLND acts as a miRNA sponge to positively regulate mRNA expression in a competing endogenous RNA (ceRNA)-dependent manner, we selected SKCM cells for *in-vitro* experiments. First, we determined that the expression level of GRASLND was significantly reduced after transfection with shGRASLND (Fig. 6A), the expression level of miR-218-5p was also significantly decreased after transfection with the miR-218-5p inhibitor (Fig. 6B), and the expression level of STAM2 was significantly increased after transfection with STAM2 OE (Fig. 6C). In SKCM cells, miR-218-5p attenuated the effect of GRASLND. shGRASLND in A2058 and SK-MEL-28 cells resulted in significantly greater miR-218-5p expression and significantly lower STAM2 mRNA expression (Fig. 6D-F). A2058 and SK-MEL-28 cells were transfected with shNC, shGRASLND, or shGRASLND in combination with the miR-218-5p inhibitor or with the STAM2 OE to investigate the effect of miR-218-5p inhibition on STAM2 expression. shGRASLND reduced STAM2 expression, while the miR-218-5p inhibitor partially inhibited this effect (Fig. 6D-F). Given that GRASLND can inhibit miR-218-5p expression, we investigated whether miR-218-5p affects the GRASLND knockdown-mediated inhibition of SKCM cell progression. As expected, we observed that the downregulation of miR-218-5p expression abrogated the shGRASLND-induced reduction in A2058 cell migration and invasion (Fig. 6G-J). Compared with those of cells transfected with shGRASLND alone, the migration and invasion of A2058 cells were significantly greater after cotransfection with the miR-218-5p inhibitor and shGRASLND (Fig. 6G-J). Similarly, the migratory and invasive abilities of A2058 cells were significantly greater after cotransfection of STAM2 OE and shGRASLND than after transfection with shGRASLND alone (Fig. 6G-J). These results suggest that the downregulation of GRASLND expression inhibits SKCM invasion and migration by negatively regulating miR-218-5p expression and that GRASLND can act as a miR-218-5p sponge to positively regulate STAM2 expression.

## Discussion

Skim cutaneous melanoma is one of the most life-threatening skin cancers worldwide and one of the most common malignant tumors [26, 27]. However, the molecular mechanisms underlying its malignant behavior are poorly understood. LncRNAs are involved in various cellular processes and have been implicated in the development and progression of malignant tumors [28–31]. In the present study, we screened for a key lncRNA, GRASLND, which is associated with SKCM immunotherapy, promotes SKCM metastasis, and is significantly correlated with SKCM malignant behavior, disease-specific survival, OS, and PFS rate. In addition, GRASLND expression is associated with immune infiltration and immunotherapy outcomes in SCKM patients. Our findings will potentially lead to the discovery of new candidate markers for the immunization against and treatment of SKCM.

Little is known about GRASLND, which is located on human chromosome 2p25.1 and plays a key role in the malignant progression of tumors [32–34]. Increased expression of GRASLND in gastric cancer tissues has been reported to promote tumor metastasis and proliferation by targeting the miR-30c-2-3p/LOX axis, suggesting a strong correlation between GRASLND, poor prognosis, and advanced disease in SKCM patients [33]. In addition, GRASLND promotes mesenchymal stem cell chondrogenesis by inhibiting the interferon type II signaling pathway [35]. Furthermore, GRASLND promotes bladder cancer progression through the miR-455-5p/SOX11 axis [36]. In our study, we revealed increased expression of GRASLND in SKCM tissues, suggesting a strong correlation between GRASLND, poor prognosis, and immunotherapy in SKCM patients. In addition, functional experiments confirmed the promotional effect of GRASLND on SKCM migration and invasion. We also found mutual inhibition of GRASLND and miR-218-5p expression, suggesting that GRASLND has a miR-218-5p-mediated tumor suppressor effect on SKCM cells. We also demonstrated that GRASLND acts as a miR-218-5p sponge to positively regulate STAM2 expression in SKCM cells. Thus, our results demonstrate the oncogenic role of GRASLND in the malignant process of SKCM.

The ceRNA mode is a characteristic way for lncRNAs to participate in gene regulation, whereby lncRNAs competitively bind to miRNAs to eliminate the endogenous repressive effects of miRNAs on their target transcripts [37, 38]. In the present study, GRASLND was suggested to act as a ceRNA by binding to miR-218-5p. It has been shown that miR-218-5p is associated with the regulation of proliferation and migration through the function of specific target genes [39–41]. For example, miR-218-5p in the endometrial microenvironment prevents the migration of ectopic endometrial stromal cells by inhibiting LASP1 [42]. miR-218-5p regulates skin and hair follicle development by targeting SFRP2 through the Wnt/β-catenin signaling pathway [43]. miR-218-5p targets LHFPL3 to regulate human glioma cell proliferation, migration, and epithelial–mesenchymal transition [44]. Similarly, we found that downregulation of GRASLND significantly increased miR-218-5p levels. Subsequently, by predicting potential miR-218-5p targets via TargetScan, miRTarBase, and miRDB, we screened STAM2 as a target of miR-218-5p. STAM2 is a phosphotyrosine protein that is a member of the endosomal sorting complex required for transport (ESCRT-0) [45, 46]. Kaymaz et al. revealed that STAM2 is a functional target of miR-2278, which acts as a tumor suppressor by inhibiting leukemia cell proliferation and inducing apoptosis [47]. STAM2 knockdown has been reported to inhibit proliferation, migration, and invasion by affecting the JAK2/STAT3 signaling pathway in gastric cancer [46]. Our rescue experiments further confirmed that overexpression of STAM2 reversed the effects of miR-218-5p on cells in vitro. Moreover, most lncRNAs function by interacting with various proteins [48]. Similarly, in SKCM, considering its localization, GRASLND may interact with STAM2 proteins and alter gene expression or participate in gene regulation as a cofactor of transcription factors and chromatin modifiers [49, 50]. These data suggest that miR-218-5p regulates STAM2 expression in SKCM, thus regulating SKCM cell function.

In conclusion, our study highlights the value of GRASLND as a prognostic marker or therapeutic target for SKCM. GRASLND predicts poor prognosis in SKCM patients and is correlated with tumor metastasis. In addition, GRASLND promoted SKCM invasion and migration by regulating STAM2 expression through the competitive uptake of miR-2218-5p. One of the shortcomings of this study is that no animal experiments were performed, and in vivo experiments are essential; therefore, we will perform these experiments in the future.

## Data Availability

The datasets used and/or analysed during the current study are available from the corresponding author on reasonable request.
